# Uneven Landscape of Cognitive–Motor Dual-Task Research Across Rehabilitation Fields: A State-of-the-Field and Pattern Analysis

**DOI:** 10.1016/j.arrct.2026.100597

**Published:** 2026-02-05

**Authors:** Jun Woo Kwon

**Affiliations:** Department of Physical Education, Seoul National University, Seoul, Republic of Korea

**Keywords:** Cognitive–motor dual-task, Conceptual analysis, Geriatric and neurologic rehabilitation, Interdisciplinary imbalance, Rehabilitation, Rehabilitation disparities

## Abstract

Cognitive–motor dual-task (CMDT) research has expanded rapidly over the past 2 decades, yet this growth has occurred almost exclusively within geriatric and neurologic rehabilitation. In contrast, musculoskeletal, sports, cardiopulmonary, pediatric, and pain rehabilitation fields demonstrate markedly limited engagement with CMDT despite its relevance to motor control and functional performance. This Special Communication synthesizes the current state-of-the-field and examines the structural mechanisms that have produced this persistent interdisciplinary imbalance. Drawing from existing literature, the analysis identifies a set of recurring patterns: (1) CMDT emerged historically within domains where gait, balance, and cognitive decline are central clinical outcomes, creating an early advantage for geriatric and neurologic research; (2) CMDT-sensitive outcomes are not equally prioritized across all rehabilitation fields, reducing its perceived relevance in others; (3) standardization of dual-task assessments has developed primarily within neurologic and aging research, limiting methodological accessibility elsewhere; (4) clinician-level barriers—including time, expertise, and tool availability—restrict CMDT implementation even within well established settings; and (5) evidence and expertise have accumulated most rapidly in the fields where CMDT was first adopted, reinforcing its continued concentration in those domains. Collectively, these patterns suggest that the uneven distribution of CMDT research is not a transient artifact but a reflection of deeper disciplinary structures within rehabilitation science. By clarifying these mechanisms, this work provides a conceptual foundation for understanding why CMDT has flourished in some fields but not others, without presuming uniform applicability or advocating for indiscriminate expansion.

## Highlights

• Cognitive–motor dual-task (CMDT) research is concentrated in neurologic and geriatric rehabilitation.
• Uneven CMDT growth reflects structural differences across rehabilitation fields.
• Outcome sensitivity shapes whether CMDT is clinically central or peripheral.
• Standardized CMDT metrics lower entry barriers and accelerate evidence synthesis.
• Implementation barriers persist even in CMDT-mature settings (time, tools, training).

Cognitive–motor dual-task (CMDT) describes performing a cognitive activity during a motor task, which competes for limited attentional and executive resources. CMDT reliably produces performance decrements—including slower reaction times, reduced gait velocity, and diminished postural stability—across multiple studies.^1^, ^2^, ^3^ These effects are especially pronounced in older adults and individuals with neurologi impairments: adding cognitive load during walking increases fall likelihood, whereas CMDT-oriented training can improve balance, cognitive abilities, and gait performance.^3^^,^^4^ Consequently, CMDT has become a core component of evaluation and therapeutic planning in neurologic and geriatric rehabilitation.

In contrast, musculoskeletal (MSK), sports, cardiopulmonary, pediatric, and chronic pain rehabilitation rarely incorporate CMDT principles. These specialties prioritize biomechanical restoration, physical performance, physiological capacity, developmental considerations, or pain management rather than cognitive–motor coupling, resulting in marked disciplinary disparities.

Field-specific clinical relevance of CMDT outside neurologic/geriatric care is increasingly apparent. In MSK and postsurgical rehabilitation, patients often return to community ambulation and sport under distraction, where attentional load can expose residual movement-control deficits and reinjury risk (eg, cutting/landing decisions after anterior cruciate ligament (ACL) reconstruction). In cardiopulmonary rehabilitation, daily walking commonly occurs under symptom monitoring and pacing decisions; concurrent cognitive demands may amplify dyspnea-related instability and compromise safety behaviors. In pediatric rehabilitation, dual-task demands are embedded in school and play contexts where motor performance must be coordinated with instruction-following and executive control, requiring developmentally calibrated paradigms. In chronic pain, attentional capture by pain and fear-avoidance may interact with motor control under distraction, making CMDT a potential probe of functional vulnerability rather than a universal training target.

This article therefore aimed to: (1) provide an updated overview of CMDT research deployment across rehabilitation domains; (2) characterize field-specific developmental patterns; and (3) identify documented research gaps. Although CMDT’s prominence originates largely from neurologic and geriatric contexts, many real-world and sport-specific activities in other populations also require simultaneous cognitive regulation and motor control (eg, rapid direction changes after ACL reconstruction, ambulation under dyspnea monitoring, or navigating school environments during instruction). Whether current CMDT paradigms are conceptually suited or practically adaptable to these contexts remains unclear, and the potential value of field-tailored dual-task assessment or training has been insufficiently explored. The primary focus of this article is CMDT assessment and research deployment patterns across fields; training is discussed only insofar as it informs assessment paradigms and translational pathways. Across these fields, CMDT can function as an ecologically valid “stress test” to reveal performance vulnerabilities that may remain undetected under single-task evaluation.

## Historical and theoretical background of cognitive–motor dual-task research

### Theoretical foundations of the dual-task concept

Dual-task research originates from the long-standing premise in cognitive psychology that attention and information-processing capacity are limited. Foundational theories such as Kahneman’s capacity-limited attention model help explain why motor execution may deteriorate when a concurrent cognitive task is introduced.^5^ Within this framework, gait and postural control are viewed not as purely automatic behaviors but as actions requiring active cognitive regulation. Consistent with this understanding, empirical work has shown that imposing cognitive demands while walking results in slower speeds, increased gait variability, and compromised balance, particularly in older adults.^6^

More contemporary accounts have expanded beyond a single shared-resource model. Capacity-sharing and bottleneck theories distinguish situations in which tasks compete for identical processing stages from those in which decrements reflect strategic reallocation of attention.^7^^,^^8^ In gait-related CMDT, this conceptual diversification has informed principles such as “posture-first”—a shift toward stability when balance is threatened—and “cognition-first” in low-threat environments.^9^ Executive control perspectives further propose that dual-task gait costs arise when limited frontal resources must coordinate competing goals, update sensory inputs, and suppress interference.^10^ Neuroimaging evidence supports these models, linking high dual-task gait costs to altered activity and connectivity within prefrontal and frontoparietal networks, especially in older adults and individuals with neurodegenerative disease.^11^ Collectively, these perspectives frame CMDT as a lens for examining how executive systems manage concurrent motor and cognitive demands.

### Early development within neurologic and geriatric rehabilitation

CMDT became clinically important in populations where cognitive decline and gait impairment were already central therapeutic concerns—namely neurologic and aging-related groups. A meta-analysis published in 2017 demonstrated that even simple cognitive tasks (eg, verbal fluency, mental arithmetic) reliably disrupt walking performance and significantly reduce gait speed in older adults.^12^ Early pilot randomized controlled trials in stroke rehabilitation also showed that dual-task gait practice produced greater combined cognitive–motor gains than motor-only gait training.^13^ These findings established attentional load as a meaningful determinant of motor behavior and positioned CMDT early as an essential construct in neurologic and geriatric rehabilitation.

### Resulting differences in the starting point across rehabilitation fields

Because these fields adopted CMDT early, they rapidly accumulated theoretical frameworks, standardized metrics (eg, dual-task cost [DTC], dual-task gait speed), and clinical expertise concerning cognitive–motor interaction.^6^ In contrast, MSK and sports rehabilitation evolved around principles of biomechanical restoration, tissue healing, and performance optimization, in which cognitive load was not initially viewed as a primary determinant of movement outcomes. Consequently, dual-task paradigms were not embedded into their formative research trajectories.

Taken together, these historical and theoretical differences shaped the uneven developmental baseline from which CMDT expanded—a pattern that underlies the field-specific disparities analyzed in later sections.

## State-of-the-field overview: current distribution of cognitive–motor dual-task research across rehabilitation fields

### Scoping search to characterize the field

A summary of domain-specific CMDT counts derived from this scoping process is presented in table 1.

**Table 1:** Distribution of CMDT-related literature identified in the scoping search (2010-2025).

Rehabilitation Domain	N Studies	% of Total	Inclusion Filters
Neurologic/geriatric	2081	82.60	Parkinson, stroke, dementia
Musculoskeletal (MSK)	31	1.20	ACL, osteoarthritis, CAI
Sports	75	3.00	Athlete, football, sport*
Cardiopulmonary	12	0.50	COPD, heart failure
Pediatric	216	8.60	Children, ADHD, TBI
Chronic pain	32	1.30	Chronic low back pain, fibromyalgia
Total	2519	100	–

Percentages reflect the share of records after hierarchical assignment to a single primary rehabilitation domain. Although records may match multiple domain-specific terms during initial retrieval and filter sensitivity varies by field, counts should be interpreted as comparative indicators of field engagement rather than exact prevalence estimates.

TBI, traumatic brain injury.

⁎ indicates truncation in the search strategy (e.g., sport, sports, sporting).

A targeted scoping search (2010-2025) was conducted to map the relative distribution of. CMDT publications across rehabilitation domains rather than to perform a comprehensive systematic review. Records were retrieved using core CMDT terms combined with field-specific filters (table 1) and were then assigned to a single primary domain using a hierarchical rule to avoid double counting when studies spanned multiple areas. Counts therefore represent an approximate landscape indicator of field engagement, sensitive to filter breadth and indexing practices.

### Overall CMDT publication landscape

Research on CMDT performance has expanded in recent years, but its distribution remains uneven across rehabilitation fields, as reflected in the scoping summary presented in table 1.

Neurologic and aging-related CMDT research is distinguished by consistent empirical evidence for dual-task interference. Smith et al^12^ showed that adding a cognitive task reduces gait speed and increases variability in older adults. Heselton et al^14^ confirmed significant dual-task–related slowing in walking speed. Mustafovska et al^15^ likewise demonstrated elevated proportional dual-task costs across gait and cognitive domains. Collectively, these findings support the clinical utility of CMDT outcomes and explain their central role in neurologic and geriatric rehabilitation.

Systematic reviews and meta-analyses synthesizing CMDT findings are increasingly common in these domains.^3^^,^^16^ In contrast, analogous review-level work is scarce in MSK, sports, pediatric, cardiopulmonary, and chronic pain rehabilitation, indicating that these areas remain at preliminary stages of CMDT engagement.

### Field-specific CMDT development patterns

The purpose of the field-specific examples below is not to exhaustively summarize all CMDT outcomes studied in each domain, but to illustrate dominant developmental patterns (eg, outcome focus, protocol families, and stage of evidence accumulation).^3^^,^^16^

Examples were selected to represent commonly used motor tasks (primarily gait and balance), frequently reported summary metrics (eg, DTC), and the highest-level syntheses available within each field.

### Neurologic and geriatric rehabilitation

Neurologic and geriatric rehabilitation constitute the most extensively developed fields for CMDT research. Strong links between cognitive load and gait control have been repeatedly demonstrated. Smith et al^12^ showed that gait speed and variability declines under cognitive challenge. Ramírez and Gutiérrez^6^ supported the clinical relevance of these findings by showing that dual-task gait performance predicts future cognitive deterioration. CMDT studies targeting fall prevention and brain–behavior mechanisms continue to expand; for example, Weng et al^4^ presented a meta-analysis demonstrating benefits of motor–cognitive training in individuals with dementia or mild cognitive impairment. The conceptual integration of “gait/balance performance with cognitive signal processing” has therefore become foundational in this field. In these domains, CMDT outcomes extend beyond gait speed and variability to include balance, turning, dual-task timed Up and Go, cognitive task accuracy, and neurobehavioral predictors; however, gait-based metrics remain the most standardized and comparable across studies.

### Musculoskeletal rehabilitation

MSK rehabilitation has remained grounded in biomechanical frameworks emphasizing joint mobility, muscle strength, and pain reduction. Although conceptual discussion and preliminary work exist—Al-Yahya et al^17^ suggested CMDT relevance in assessing gait and balance among MSK populations—rigorous randomized trials are scarce. CMDT remains in an early exploratory phase.

### Sports rehabilitation

Sports rehabilitation typically views cognitive load as a negative influence on agility or skill execution. Nonetheless, recent work explores CMDT’s potential. Friebe et al^18^ found that a dual-task agility measure differentiated athletic capabilities more effectively than traditional markers. Still, CMDT training is rarely incorporated into structured return-to-sport programs.

### Cardiopulmonary rehabilitation

Cardiopulmonary research has focused predominantly on physiological markers such as VO_2_ and ventilation thresholds. CMDT work remains limited. Hassan et al^19^ demonstrated changes in prefrontal oxyhemoglobin concentration during CMDT in patients with chronic obstructive pulmonary disease. Yet most studies are small and descriptive, and clinical implications remain unclear.

### Pediatric rehabilitation

Developmental heterogeneity challenges the standardization of CMDT in pediatric contexts. Manicolo et al^20^ showed altered gait patterns in children with attention-deficit/hyperactivity disorder (ADHD) under dual-task conditions. However, methodological inconsistency—age ranges, task design, measurement tools—prevents synthesis into field-wide models. CMDT remains intermittent rather than integrated.

### Pain and chronic pain rehabilitation

Although cognitive–emotional contributors to pain are well recognized, CMDT-specific evidence is sparse. Hamacher et al^21^ reported increased gait variability in chronic low back pain during cognitive dual tasks. However, nearly all studies are single session, and interventional application has not been developed.

## Gap identification: underdeveloped areas and missing research links

Outside neurologic and geriatric rehabilitation, several areas of rehabilitation science still display significant limitations in both the volume and depth of CMDT inquiry. The gaps summarized below reflect current evidence constraints in each discipline, without inferring root causes.

### Musculoskeletal rehabilitation: lack of systematic and cohesive evidence

Although CMDT studies involving individuals with MSK conditions do exist, the evidence base remains fragmented. Shi et al^22^ examined dual-task–related gait disruptions in patients after ACL reconstruction; however, its small sample size and narrow clinical representativeness limit generalizability. Similarly, a scoping review targeting patients with knee osteoarthritis reported that most investigations were constrained by small sample sizes, inconsistent cognitive task designs, and heterogeneous measurement approaches, preventing unified interpretation.^23^ Supporting reviews in chronic ankle instability populations indicate the possibility of dual-task training benefiting static and dynamic balance; however, the number of randomized trials is still very small, heterogeneity across protocols remains high, and durability of improvements has not been established.^24^^,^^25^

### Sports rehabilitation: CMDT used mainly as an evaluative rather than interventional concept

Growing attention has been directed toward motor–cognitive agility assessment in athletic contexts. For elite soccer players, a dual-task agility measure appeared to better capture on-field performance differences compared with conventional reaction testing.^18^ Nonetheless, such studies primarily validate test usefulness and rarely address rehabilitation effects after injury. A recent systematic review reported that although athletes frequently show performance deterioration under dual-task conditions, only a limited number of studies have evaluated structured CMDT-based training programs,^26^ and substantial methodological heterogeneity continues to hinder cross-study comparison.

### Cardiopulmonary rehabilitation: limited research with uncertain clinical translation

CMDT is only beginning to emerge as a research interest in cardiopulmonary settings. A study focusing on populations with chronic obstructive pulmonary disease showed that adding cognitive load led to greater reductions in gait speed, impaired balance, and pronounced cognitive interference compared with those without pulmonary disease.^27^ Yet, most available studies employ cross-sectional or small comparative designs, leaving major clinical questions unanswered—notably whether CMDT performance predicts hospitalization risk, long-term prognosis, or patient-centered outcomes such as quality of life.

### Pediatric rehabilitation: lack of developmentally aligned CMDT frameworks

Several studies have examined dual-task gait in children with ADHD, traumatic brain injury, or developmental conditions; however, the field lacks standardized developmental criteria to guide CMDT design. For example, research comparing ADHD and typically developing children showed shifts in gait patterns under dual-task conditions, with motor-weighted tasks producing the greatest influence on walking mechanics.^20^ A systematic review reinforced that gait variability increases under cognitive–motor challenge in ADHD, but also emphasized large inconsistencies in age ranges, developmental profiles, and measurement tools, preventing broad clinical generalization.^28^ Likewise, traumatic brain injury–related studies confirm that task complexity moderates dual-task interference, yet translation into routine practice has not followed.^29^

### Chronic pain rehabilitation: scarcity of integrated motor–cognitive intervention research

Although the relationship between cognition, emotion, and pain processing has long been recognized in chronic pain rehabilitation, empirical work specifically investigating dual-task motor performance is still sparse. Studies in individuals with chronic low back pain have reported altered trunk control and impaired stability while walking under cognitive distraction.^30^ Nevertheless, most research in this area remains single session and observational, offering virtually no evidence regarding whether CMDT-based rehabilitation alleviates pain or restores functional capacity.

## Field-level conceptual review: structural and disciplinary factors explaining the imbalance

The uneven concentration of CMDT research within neurologic and geriatric rehabilitation reflects not only historical patterns but also deeper structural features of rehabilitation science. Unlike section “State-of-the-field overview: current distribution of cognitive–motor dual-task research across rehabilitation fields,” which summarized the distribution of studies across fields, this section examines why CMDT became foundational in some disciplines but remained peripheral in others, focusing on paradigmatic alignment, outcome sensitivity, measurement infrastructure, clinical implementation constraints, and research-network path dependency.

To reduce subjectivity, barriers were synthesized using an implementation-oriented lens and grouped into 4 recurring categories: (1) protocol design complexity (task choice, prioritization instructions); (2) measurement and infrastructure constraints; (3) feasibility (time, staffing, training); and (4) safety and appropriateness considerations. Illustrative evidence for these categories is provided in the sections below, where clinician surveys, applied guidance papers, and technology-implementation reports are summarized.^31^, ^32^, ^33^

### Disciplinary paradigms: CMDT as a “central explanatory model” vs a “context-dependent modifier”

Earlier meta-analyses and reviews consistently reported that studies concerning cognitive load effects on gait and balance were conducted mainly in populations with neurologic disease or age-related decline (eg, Parkinson disease or prodromal dementia), showing marked deterioration in gait speed, variability, and balance.^10^^,^^17^^,^^34^ Al-Yahya et al^17^ identified strong relationships between dual-task gait performance and both aging and cognitive impairment. Leone et al^34^ emphasized the dominant focus on neural mechanisms underlying gait–balance under cognitive demand, and Li et al^10^ similarly described age-linked increases in cognitive involvement in gait control as justification for dual-task models. Wang et al^35^ additionally reported short-term fall risk reduction through cognitive–motor interference (CMI)–based interventions in older adults.

This finding aligns with a growing body of evidence demonstrating the clinical relevance of dual-task gait. Specifically, dual-task gait may serve as an early indicator of dementia progression, and dual-task metrics such as DTC have been associated with neuroanatomical changes and functionally meaningful outcomes, including cognitive decline in Parkinson disease.^11^^,^^36^ Oppositely, reviews repeatedly observed that MSK, cardiopulmonary, sports, pediatric, and pain populations are rarely included or substantially under-represented.^6^^,^^17^^,^^34^^,^^37^^,^^38^

Rather than repeating field-level summaries, the implication here is structural: CMDT aligns strongly with the core mechanisms and clinical priorities of neurologic/geriatric care but not with the guiding paradigms of other rehabilitation domains, which are grounded in biomechanics, pain science, cardiopulmonary physiology, or developmental trajectories.

### Outcome sensitivity: where CMDT meaningfully alters key outcomes

In aging and neurologic care, clinically central outcomes—such as gait speed, balance stability, fall occurrence, and cognitive performance—are highly responsive to dual-task interference.^10^^,^^12^^,^^17^^,^^35^ Dual-task gait has even been proposed as a predictor of cognitive decline,^6^ and specific motor signatures such as reduced arm swing in Parkinson disease during dual-task walking reflect cognitive dysfunction risk.^36^ Positive fall prevention effects are likewise documented.^35^

In contrast, MSK, sports, cardiopulmonary, and pain rehabilitation emphasize outcomes (pain, joint mechanics, aerobic capacity, symptom burden) that show comparatively limited dual-task responsiveness—explaining why CMDT naturally occupies different levels of centrality across fields.

### Measurement accessibility and standardization

Validated metrics—dual-task gait speed, spatiotemporal variability, DTC—are widely recognized across neurologic/geriatric CMDT research.^12^^,^^17^^,^^34^^,^^35^^,^^38^ Dual-task gait evaluation has become a normative laboratory approach.^10^ These measures show superior sensitivity over single-task gait tests for detecting subtle deficits.^6^^,^^10^^,^^17^^,^^21^^,^^34^^,^^37^

Other rehabilitation fields lack comparable standardization, creating a methodological entry barrier: inconsistent cognitive tasks, variable motor tasks, unclear prioritization instructions, and incompatible outcome definitions. Technology-supported CMDT research introduces further heterogeneity.^31^

Thus, the gap is not only historical but infrastructural—most fields lack a stable measurement framework to adopt.

### Implementation barriers within clinical environments

Implementation barriers have been explicitly documented even in CMDT-mature fields, most clearly through clinician surveys and applied guidance papers, which consistently identify time burden, protocol uncertainty, limited validated resources, and training needs as barriers.^32^^,^^33^

A survey of Parkinson clinicians identified practical challenges such as insufficient time, unclear task design, lack of validated resources, and limited confidence.^32^ A follow-up study highlighted requests for practical case scenarios and ready-to-use guidelines.^33^ Technology-supported CMDT can improve dual-task gait and cognition but requires costly equipment and additional staff training.^31^

These barriers, already present in CMDT-mature environments, likely exert even stronger suppressive effects in fields where CMDT is not culturally established.

### Research ecosystem path dependency

Major meta-analyses consistently show that CMDT research samples are dominated by older adults with neurologic conditions.^6^^,^^12^^,^^17^^,^^34^^,^^35^

Wang et al^35^ similarly remarked that CMI-based intervention trials are short-term and nearly entirely centered on elderly subjects.

Ongoing expansions in neural-mechanistic work,^11^^,^^36^ methodological reviews,^10^^,^^33^^,^^34^ and technology-focused interventions^31^ continue to reinforce this concentration.

Because research credibility, funding, and methodological norms grow where prior evidence is strongest, CMDT has remained locked into its original disciplinary home, slowing its development elsewhere.

### Structural synthesis: why imbalance persists across rehabilitation specialties

The persistence of this asymmetry stems from 5 intersecting determinants: (1) CMDT aligns directly with disease mechanisms in specific fields;^17^^,^^32^, ^33^, ^34^, ^35^ (2) only certain outcomes—gait, balance, cognitive function—strongly reflect DTCs;^6^^,^^10^^,^^12^^,^^17^^,^^32^^,^^35^ (3) standardized CMDT measurement infrastructure is unevenly distributed;^10^^,^^12^^,^^31^^,^^33^^,^^34^ (4) practical barriers impede integration even where CMDT is clinically valued;^31^, ^32^, ^33^ (5) research ecosystems reinforce path dependency and concentration in original domains.^11^^,^^17^^,^^31^, ^32^, ^33^, ^34^, ^35^

Together, these features explain why CMDT maintains a central role in neurologic and geriatric rehabilitation yet remains underdeveloped elsewhere.

### Diversity of dual-task protocols and implications for other fields

Existing CMDT work has relied on a relatively small set of protocol “families.” In neurologic and geriatric research, the most common paradigms pair level-ground walking or timed Up and Go tests with serial subtraction, verbal fluency, or simple working-memory tasks, and quantify performance using DTC in gait speed, variability, or error rate. Technology-enhanced variants use virtual reality environments, instrumented walkways, or exergaming platforms to deliver similar cognitive loads.

For translation into other rehabilitation disciplines, these paradigms have mixed suitability. For example, standard gait-plus-subtraction tasks may map reasonably well onto community ambulation demands in cardiopulmonary and chronic pain populations, provided that intensity is titrated to cardiorespiratory or symptom limits. In contrast, the same protocols may be poorly aligned with sport-specific challenges, where rapid visuomotor decision-making, opponent tracking, and ball control are more relevant than arithmetic accuracy. Here, dual-task agility tests that combine cutting, jumping, or change-of-direction drills with reactive decision tasks or visual search demands are likely more ecologically valid. Pediatric populations raise additional considerations: task difficulty and instructions must be developmentally appropriate, and playful or game-like interfaces may be essential for engagement.

These differences suggest that CMDT protocols cannot simply be “copied and pasted” from neurologic/geriatric research into other fields. Instead, protocol families should be selected or adapted according to the dominant real-world motor–cognitive challenges in each discipline, while still adhering to basic reporting standards (eg, clear description of cognitive task type, motor task, prioritization instructions, and performance metrics).

## Discussion: integrative interpretation of patterns and research imbalance

This study synthesized evidence showing that CMDT research has become deeply rooted within neurologic and geriatric rehabilitation, whereas development in MSK, sports, cardiopulmonary, pediatric, and chronic pain rehabilitation remains comparatively limited. In this section, the findings from earlier analyses are integrated to clarify the structural mechanisms shaping this uneven disciplinary landscape within rehabilitation science.

Importantly, the argument for examining CMDT outside neurologic and geriatric rehabilitation is not that all patients in these other fields should routinely undergo dual-task training. Rather, many of the core clinical problems in MSK, sports, cardiopulmonary, pediatric, and pain rehabilitation—including return-to-sport decision-making, exertional intolerance, school and community participation, and pain-related movement avoidance—unfold under conditions of cognitive load. In such contexts, dual-task paradigms could provide (1) more ecologically valid stress tests of motor control, (2) sensitive probes of executive or attentional constraints on performance, and (3) a structured way to integrate cognitive and motor targets when appropriate. Clarifying where and how CMDT adds value therefore matters for these disciplines, even if its eventual role remains more selective than in neurologic and geriatric care.

This paper distinguishes CMDT as an assessment paradigm (stress-testing motor control under cognitive load) from CMDT as a training strategy, which entails different objectives, dosing, and safety considerations.

### Field differences in CMDT adoption reflect structural patterns rather than simple variation in interest

As outlined in sections “State-of-the-field overview: current distribution of cognitive–motor dual-task research across rehabilitation fields” and “Gap identification: underdeveloped areas and missing research links,” CMDT research shows markedly uneven development across rehabilitation fields. These field-specific trends align closely with paradigm and outcome characteristics identified in section “Discussion: integrative interpretation of patterns and research imbalance.” Within aging and neurologic populations, outcomes such as gait, balance, fall risk, and cognitive function are strongly influenced by dual-task interference, and CMDT performance is regarded as an early indicator of functional or cognitive decline supported by robust data.^17^^,^^34^^,^^36^ By contrast, other rehabilitation disciplines historically emphasize different endpoints-pain reduction, biomechanical integrity, physiological load tolerance, or developmental milestones-variables that show weaker responsiveness to dual-task effects. In those domains, CMDT consequently remains peripheral rather than foundational. Therefore, the distinct developmental trajectories of CMDT across fields arise from conceptual and structural differentiation within rehabilitation science—not merely from varied interest levels.

### The CMDT research ecosystem exhibits path dependency formed around early paradigmatic dominance

Section “Discussion: integrative interpretation of patterns and research imbalance” highlights that CMDT’s evidence base expanded earliest in a small set of rehabilitation domains, creating path-dependent growth that continues to shape where new syntheses and mechanistic studies accumulate.^6^^,^^17^^,^^34^^,^^35^ Subsequent expansions—systematic reviews, mechanistic neuroimaging work, and studies using technology-supported interventions^11^^,^^31^^,^^36^^,^^37^—have largely remained inside this same disciplinary cluster. Where CMDT research is sparse (eg, MSK, sports, cardiopulmonary, pediatric, pain), the lack of established infrastructure restricts the development of randomized controlled trials and long-term intervention trials, reinforcing a persistent cycle of limited evidence accumulation. As a result, CMDT research advancement demonstrates path dependency, maintaining the dominance of its original clinical sectors.

### Standardization of measurement tools determines entry barriers to CMDT research

Standard evaluation tools such as dual-task gait speed, DTC, and timed Up and Go dual-task are widely adopted in aging and neurologic fields, providing a shared methodological framework that accelerates research and supports consistent clinical implementation.^17^^,^^32^, ^33^, ^34^, ^35^ However, many other rehabilitation specialties lack agreement on fundamental design elements—including cognitive task type, motor task selection, difficulty progression, and selected endpoints. This methodological heterogeneity reduces feasibility and slows uptake.^31^^,^^33^ Thus, the presence or absence of validated and accessible measurement standards constitutes a major determinant of CMDT adoption across disciplines.

### Barriers related to clinical implementation, training, time, and expertise limit CMDT diffusion

As detailed in section “Discussion: integrative interpretation of patterns and research imbalance,” clinical professionals cite practical constraints—such as insufficient available time, difficulty in designing or preparing dual-task protocols, a shortage of validated tools, and limited confidence in delivering CMDT effectively—as reasons for low adoption.^31^, ^32^, ^33^ These barriers occur even in neurologic and geriatric contexts where CMDT is already well established, suggesting that in fields where CMDT remains peripheral, implementation challenges may present even greater obstacles. Consequently, translation of CMDT evidence into real-world practice is not automatic; professional workload, training structures, and environmental limitations all reinforce field-specific disparities.

### CMDT is not a universally transferrable paradigm across all rehabilitation domains

Sections “Gap identification: underdeveloped areas and missing research links” and “Field-level conceptual review: structural and disciplinary factors explaining the imbalance” highlighted pronounced differences across rehabilitation specialties in terms of underlying pathophysiology, prioritized outcomes, operational constraints, and population characteristics. These differences indicate that successful CMDT applications in one field cannot simply be transposed into another without adaptation. Although CMDT can function as a central paradigm where CMI directly impacts meaningful clinical change (eg, aging, neurologic rehabilitation), fields guided by alternative goals require tailored design and theoretical justifications—not uniform expansion. Thus, disparities in CMDT development also reflect structural compatibility differences rather than solely uneven progress.

### The field-specific imbalance in CMDT reflects structural characteristics of rehabilitation science

Across previous sections, CMDT disparities appear rooted in the combined influences of (1) paradigm distinctions, (2) differential outcome sensitivity, (3) uneven measurement standardization, (4) clinical implementation challenges, and (5) path-dependent evidence ecosystems. Together, these factors illustrate a broader organizing principle within rehabilitation science: disciplinary paradigms shape whether a construct becomes central or remains peripheral. CMDT therefore serves as a compelling and visible example of how structural forces direct scientific and clinical evolution within rehabilitation fields.

## Field-specific recommendations and call to action

Field-specific and cross-cutting recommendations are summarized in tables 2 and 3 to support structured decision-making for future CMDT research and clinical implementation. Recommendations were derived by linking (1) the dominant protocol families and outcomes currently used in each field (sections “State-of-the-field overview: current distribution of cognitive–motor dual-task research across rehabilitation fields,” “Gap identification: underdeveloped areas and missing research links,” and “Field-level conceptual review: structural and disciplinary factors explaining the imbalance”) with (2) field-specific constraints (eg, safety limits, developmental heterogeneity, feasibility) and (3) the type of evidence currently available (descriptive studies vs trials vs syntheses). Accordingly, some items are intentionally cross-cutting (table 3), whereas table 2 emphasizes how those general principles require different operationalization across domains.

**Table 2:** Recommended CMDT priorities across rehabilitation fields.

Field	Key Recommendations
Neurologic & Geriatric Rehabilitation	(1) Shift from demonstrating dual-task costs to refining mechanisms and harmonizing outcome measures
	(2) Prioritize multicenter efforts to establish a core CMDT outcome set, including standardized instructions, prioritization rules, and minimal reporting requirements
Musculoskeletal & Sports Rehabilitation	(1) Avoid direct transfer of neurologic paradigms; instead identify field-relevant motor–cognitive situations (eg, cutting with decision-making, landing under distraction)
	(2) Develop simple, clinically feasible dual-task tests tailored to these demands
	(3) Evaluate whether dual-task performance provides incremental value for return-to-sport and reinjury risk algorithms
Cardiopulmonary Rehabilitation	(1) Employ walk-based CMDT protocols that maintain physiological safety
	(2) Investigate how cognitive load affects pacing, dyspnea perception, and safety behaviors
	(3) Conduct prospective studies to assess prediction of hospitalization, exacerbations, and participation outcomes
Pediatric Rehabilitation	(1) Develop age-banded, developmentally appropriate CMDT frameworks
	(2) Provide guidance on scaling cognitive demand
	(3) Integrate CMDT tasks into play-based or school-relevant activities, ideally through collaboration with developmental psychologists and educators
Pain Rehabilitation	(1) Use CMDT to probe attentional control, fear-avoidance, and safety behaviors during movement
	(2) Before clinical adoption, determine whether reducing motor–cognitive interference yields improvements beyond psychologically informed approaches

(1) In cardiopulmonary populations, “physiological safety” refers to ensuring that dual-task walking does not exceed symptom-limited thresholds (eg, excessive dyspnea, oxygen desaturation, abnormal hemodynamic responses) and that pacing/monitoring procedures are preserved while cognitive load is introduced.

(2) In pediatric rehabilitation, age-banding is necessary because executive capacity, attention span, and instruction comprehension change rapidly with development; therefore, the same nominal cognitive task can impose qualitatively different loads across ages. Practical scaling guidance should specify how to adjust task complexity, response modality, and prioritization instructions to maintain comparable challenge across developmental stages.

**Table 3:** Cross-cutting priorities for all rehabilitation fields.

Category	Recommendations
Field-level visibility	Report simple disciplinary metrics (eg, study distribution, availability of RCTs) to make CMDT imbalances transparent
Protocol design	Codesign CMDT protocols with frontline clinicians to ensure feasibility, ecological validity, and relevance to routine care
Scope boundaries	Explicitly identify when CMDT is not expected to be central to a rehabilitation goal to prevent indiscriminate or low-yield expansion

RCT, randomized controlled trial.

## Conclusion

This review analyzed how CMDT research has developed unevenly across rehabilitation science, revealing a clear structural imbalance in its disciplinary distribution. Historically, CMDT emerged in geriatric and neurologic rehabilitation—fields in which declines in gait, balance, and cognition represent primary therapeutic targets. Because these core outcomes are highly sensitive to dual-task interference, CMDT was quickly recognized as clinically essential and rapidly institutionalized into assessment and intervention models. As a result, these domains advanced early in both theoretical foundations and methodological standardization surrounding CMDT.

In contrast, MSK, sports, cardiopulmonary, pediatric, and chronic pain rehabilitation have been guided by paradigms emphasizing outcomes such as pain modulation, biomechanical restoration, physiological endurance, or developmental progression. These indicators typically show weaker direct responsiveness to CMI, resulting in limited early adoption of CMDT. In addition, a shortage of standardized protocols and practical challenges—including restricted clinical time, insufficient professional training, and task design complexity—have further constrained diffusion of CMDT-based approaches in these areas. Consequently, research momentum has remained concentrated in the domains where CMDT originated, demonstrating a path-dependent pattern of scientific growth.

Taken together, the unequal distribution of CMDT research reflects deeper conceptual and structural differentiation within rehabilitation science rather than a simple disparity in research attention. The aim of this review was not to argue for uniform expansion of CMDT across all specialties, but to clearly delineate the mechanisms through which CMDT became a dominant paradigm in some fields and remained peripheral in others. By articulating the structural drivers of this imbalance, this work provides a foundation for evaluating where CMDT can hold meaningful clinical relevance and how its future evolution may be tailored to the diverse contexts that define modern rehabilitation practice.

## Disclosure

The investigators have no financial or nonfinancial disclosures to make in relation to this project.

## Declaration of generative AI and AI-assisted technologies in the writing process

During the preparation of this work the author used ChatGPT in order to refine grammar and language. After using this tool, the author reviewed and edited the content as needed and takes full responsibility for the content of the publication.
